# Platelet‐Mimicking Therapeutic System for Noninvasive Mitigation of the Progression of Atherosclerotic Plaques

**DOI:** 10.1002/advs.202004128

**Published:** 2021-02-18

**Authors:** Yi Ma, Yuxuan Ma, Mengqiu Gao, Zhihao Han, Wen Jiang, Yueqing Gu, Yi Liu

**Affiliations:** ^1^ State Key Laboratory of Natural Medicines School of Engineering China Pharmaceutical University Nanjing 211198 China

**Keywords:** atherosclerotic plaques, photodynamics, platelet‐mimicking systems, radionuclide imaging

## Abstract

Atherosclerotic plaque is the primary cause of cardiovascular disorders and remains a therapeutic hurdle for the early intervention of atherosclerosis. Traditional clinical strategies are often limited by surgery‐related complications or unsatisfactory effects of long‐term drug administration. Inspired by the plaque‐binding ability of platelets, a biomimic photodynamic therapeutic system is designed to mitigate the progression of atherosclerotic plaques. This system is composed of photosensitizer‐loaded upconversion nanoparticle cores entrapped in the platelet membrane. The platelet membrane coating facilitates specific targeting of the therapeutic system to macrophage‐derived foam cells, the hallmark, and main component of early stage atherosclerotic plaques, which is firmly confirmed by in vivo fluorescent and single‐photon emission computed tomography/computed tomography (SPECT/CT) radionuclide imaging. Importantly, in vivo phototherapy guided by SPECT/CT imaging alleviates plaque progression. Further immunofluorescence analysis reveals foam cell apoptosis and ameliorated inflammation. This biomimic system, which combines plaque‐binding with radionuclide imaging guidance, is a novel, noninvasive, and potent strategy to mitigate the progression of atherosclerotic plaque.

## Graphical abstract

Inspired by the early stage atherosclerotic plaque‐binding ability of platelets, a biomimic photodynamic therapeutic system is designed to accurately localize and noninvasively mitigate the progression of atherosclerotic plaques. This biomimic system synergizes plaque‐binding and radionuclide imaging guidance, providing a novel, noninvasive, and potent strategy for mitigating the progression of atherosclerotic plaque.

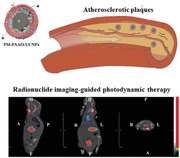

Cardiovascular disease is a disorder of the heart and blood vessels, and the leading cause of death in many parts of the world.^[^ ^1^ ^]^ Atherosclerosis is a chronic inflammatory disease affecting arteries. It is the main cause of cardiovascular disease.^[^ ^2^ ^]^ Atherosclerotic lesions are frequently found in the aorta and large aortic branches, resulting in the formation of plaques in arterial walls.^[^ ^3^ ^]^ As an atherosclerotic plaque continues to grow, it can obstruct blood flow to induce ischemic vascular diseases and even heart attack or stroke. Early intervention of atherosclerotic plaque is important. Enlarging the stenotic artery to restore blood flow is a critical factor in the treatment of atherosclerosis.^[^ ^4^ ^]^

Atherosclerotic plaque development often remains asymptomatic until a clinical event, which mostly occurs at advanced stages of atherosclerosis.^[^ ^5^ ^]^ Intravascular interventional therapies, such as endarterectomy, can rapidly enlarge the artery lumen and re‐establish blood flow. However, these invasive therapies frequently result in many complications, such as thrombosis of the endarterectomized segment and surgery‐induced myocardial infarction.^[^ ^6^ ^]^ Additionally, the use of clinical medicines like statins to improve lipid profiles and aspirin to reduce platelet clotting often require long‐term therapy and rarely achieve satisfactory lumen enlargement.^[^ ^7^ ^]^ Therefore, efforts for early intervention of atherosclerosis with a more efficient and noninvasive method are urgently needed.

At the initiation of atherosclerosis, the endothelial layer is damaged. Oxidized low‐density lipoprotein (LDL) accumulates, promoting local inflammation. Monocytes recruited through the activated endothelium differentiate into macrophages.^[^ ^8^ ^]^ These macrophages engulf lipids present in the plaque as an early event of atherosclerosis progression. This results in the formation of foam cells.^[^ ^9^ ^]^ Foam cell build‐up contributes to plaque lipid storage and sustained plaque growth. Over time, atherosclerotic plaques form with a lipid‐rich core containing foam cells covered by a fibrous smooth muscle cell cap. Plaque rupture induces thrombus formation and subsequent clinical events. Therefore, the lipid core full of foam cells derived from macrophages is a promising therapeutic target for early intervention and alleviation of atherosclerosis.^[^ ^10^ ^]^

Photodynamic therapy (PDT) induces cell death by autophagy and regulates lipid metabolism in macrophage‐derived foam cells.^[^ ^11^ ^]^ However, some obstacles remain in the clinical adoption of PDT. One notable drawback is that traditional photosensitizers, such as zinc phthalocyanine (ZnPc) and chlorin e6 (Ce6), can only be activated by visible light irradiation, which has limited penetration depth and is rarely performed in vivo.^[^ ^12^ ^]^ Lanthanide‐doped upconversion nanoparticles (UCNPs) can convert near‐infrared photons to visible wavelengths, resulting in extensive tissue penetration. Thus, the use of upconversion luminescence to activate photosensitizers in vivo^[^ ^13^ ^]^ is an attractive approach. Efficient delivery of photosensitizers to the plaque of early stage atherosclerosis is another important concern in phototherapy. Platelets are closely related to the development of atherosclerotic plaques, even at the initiation of plaque formation.^[^ ^14^ ^]^ Recently, the use of platelet membrane (PM) to identify early stage plaques by specific binding to plaque‐infiltrated macrophages was reported.^[^ ^15^ ^]^ We sought to leverage this natural binding ability to design a specific plaque‐targeting system to efficiently deliver photosensitizers to atherosclerosis sites.

In this study, we designed a PM‐coated nanoconstruct (PM‐PAAO‐UCNPs) containing UCNPs and the Ce6 photosensitizer for accurate localization of plaques and noninvasive PDT of atherosclerosis. The nanoconstruct was labeled with indocyanine green (ICG) fluorescence dye and radionuclide ^125^I to identify plaque localization in vivo and to precisely guide the therapeutic irradiation target region. The effect of PDT on atherosclerotic plaque via reactive oxygen species (ROS)‐induced apoptosis and regulated lipid metabolism was investigated in macrophage‐derived foam cells in vitro and a mouse model featuring disturbed flow‐induced carotid artery atherosclerosis in vivo.

This platelet‐mimic system was prepared by incorporating UCNPs and Ce6 into polyacrylic acid‐n‐octylamine (PAAO) micelles, followed by PM coating (**Figure** **1**a). Transmission electron microscopy (TEM) analysis revealed the formation of a distinctive hexagonal phase nanostructure and unilamellar membrane (≈10 nm) coating the PAAO‐UCNPs micelle core (Figure 1b; Figure S1a, Supporting Information), clearly verifying the PM coating. Dynamic light scattering and surface charge analyses also validated the successful membrane coating (Figure 1c,d). Meanwhile, attributable to the hydrophilic membrane cover, the stability of PM‐PAAO‐UCNPs was improved compared to the bare PAAO‐UCNPs core (Figure S1b, Supporting Information). Coomassie Brilliant Blue staining revealed identical protein profiles, indicating that PM‐PAAO‐UCNPs predictably inherited the membrane proteins from the source platelets (Figure S1c, Supporting Information) and potentially inherited the atherosclerosis‐targeting ability.

**Figure 1:**
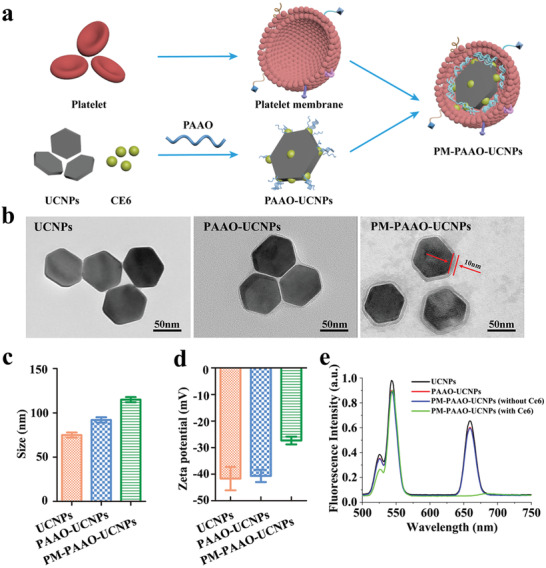
Design and characterization of PM‐PAAO‐UCNPs. a) Schematic illustration showing the composition of PM‐PAAO‐UCNPs. b) Transmission electron microscopy images of UCNPs, UCNPs loaded in micelles (PAAO‐UCNPs), and platelet member‐coated PAAO‐UCNPs (PM‐PAAO‐UCNPs). c) Size and d) zeta potential of UCNPs, PAAO‐UCNPs, and PM‐PAAO‐UCNPs in PBS. Data are presented as mean ± SD (*n* = 3). e) Fluorescence spectra of UCNPs, PAAO‐UCNPs, and PM‐PAAO‐UCNPs with or without Ce6 under 980 nm laser irradiation.

Spectral analysis of this platelet‐mimic system was further performed. Under the excitation at 980 nm, the UCNPs shows three emission bands at 514–534, 534–560, and 635–680 nm, attributable to the ^2^H_11/2_→^4^I_15/2_,^4^S_3/2_→^4^I_15/2_, and ^4^F_9/2_→^4^I_15/2_ transitions of Er^3+^ (Figure 1e; Figure S1d, Supporting Information). After the coating with PAAO and PM, the fluorescence signal remained unchanged. Since the 635–680 nm emission spectrum of UCNPs (energy donor) and the absorption band of Ce6 (energy acceptor) was overlapped, after Ce6 modified on the surface of PAAO‐UCNPs, this 635–680 nm peak disappeared through the fluorescence resonance energy‐transfer (FRET) process.^[^ ^16^ ^]^ Thus, the platelet‐mimicking system might be capable of activating the photosensitizer Ce6 with near‐infrared light (980 nm) irradiation to produce cytotoxic ROS in the treatment of atherosclerosis. To verify this hypothesis, ROS production induced by PM‐PAAO‐UCNPs in physiological solution was measured using a 1, 3‐diphenylisobenzofuran (DPBF) probe (Figure S1e, Supporting Information). When the 980 nm irradiation time increased from 0 to 20 min, the absorption of DPBF significantly decreased, indicating that this platelet‐mimicking upconversion system could produce ROS under near‐infrared irradiation.

After confirming successful nanostructure fabrication, the binding potential of PM‐PAAO‐UCNPs to different elements that play critical roles in atherogenesis was explored in vitro. Since foam cells are the major component of lipid streaks, which are the earliest lesions formed in atherosclerosis, macrophage‐derived foam cells were chosen as the target in the in vitro binding ability investigation. Foam cells were converted by incubating primary peritoneal macrophages (RAW) with oxidized LDL.^[^ ^17^ ^]^ The success of conversion was confirmed by Oil Red O staining (Figure S2, Supporting Information). Foam cells, RAW, and human vascular endothelial cells (HUVECs) were incubated with different nanostructure groups for binding analysis. Erythrocyte membrane‐coated UCNPs (EM‐PAAO‐UCNPs) (Figure S3, Supporting Information) and bare PAAO micelles (PAAO‐UCNPs) were used as controls. Unlike the controls, platelet membrane coating nanostructures PM‐PAAO‐UCNPs were readily internalized by macrophage‐derived foam cells, and the foam cells exhibited bright fluorescence (*λ*_ex_ = 980 nm, *λ*_em_ = 540 nm; **Figure** **2**a). In contrast, the fluorescent signal of PM‐PAAO‐UCNPs was barely observed in HUVECs and RAW. These results indicated that this platelet‐mimic strategy could be applied to specifically identify foam cells in atherogenesis and potentially enable the selective delivery of therapeutic agents.

**Figure 2:**
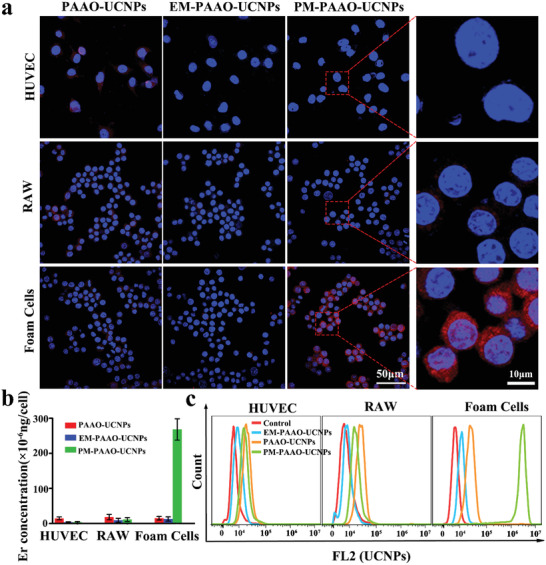
Binding ability of PM‐PAAO‐UCNPs to foam cells in vitro. a) Confocal laser fluorescence imaging of RAW, HUVEC, and foam cells incubated with PAAO‐UCNPs, EM‐PAAO‐UCNPs, and PM‐PAAO‐UCNPs, respectively. Blue and red fluorescence indicates nuclei and UCNPs, respectively. b) Quantification on the basis of Er concentration in cells. Data are presented as mean ± SD (*n* = 3). c) Flow cytometry analysis of respective affinity of PAAO‐UCNPs, EM‐PAAO‐UCNPs, and PM‐PAAO‐UCNPs to RAW, HUVEC, and foam cells.

Meanwhile, inductively coupled plasma mass spectrometry (ICP‐MS) was performed to quantify the concentration of Er^3+^ in each cell line. The results indicated that much more UCNPs were internalized into foam cells after PM coating (Figure 2b). Consistently, flow cytometry analysis further demonstrated the specific binding of PM‐PAAO‐UCNPs to foam cells (Figure 2c). These results suggested that the specific targeting of PM‐PAAO‐UCNPs to foam cells resulted from platelet membrane coating, potentially enabling early stage atherosclerotic plaque tracking and cargo delivery in vivo.

A prerequisite for the in vivo efficacy of photosensitizers is the targeted accumulation in atherosclerotic plaques. Therefore, fluorescent imaging with high resolution and radionuclide imaging with deep tissue penetration were combined to track the in vivo metabolic distribution of PM‐PAAO‐UCNPs. The near‐infrared fluorescence dye ICG and radionuclide ^125^I were used to label this nanoconstruct for in vivo imaging. ApoE‐knockout homozygous mice (ApoE^−/−^) with high levels of total plasma cholesterol were used in this study. In combination with a high‐fat diet, partial carotid ligation surgery was performed to rapidly induce robust atherosclerosis in the left carotid artery (LCA) of ApoE^−/−^ mice, as shown in **Figure** **3**a.^[^ ^18^ ^]^ One hour after intravenous injection of PM‐PAAO‐UCNPs, obvious ICG fluorescent signals were observed in the LCA of living mice. The fluorescent imaging of the isolated artery further confirmed the distinct distribution of PM‐PAAO‐UCNPs between the left and right blood vessels (Figure 3b). Hematoxylin and eosin (H&E) staining and fluorescent imaging of cross‐sections of the vessels (Figure 3c) clearly demonstrated that atherosclerotic plaques occurred in the LCA and that PM‐PAAO‐UCNPs accurately accumulated in the fat core of the plaques. These findings indicated that the PM coating strategy could enable specific tracking and accumulation at atherosclerotic plaques. More importantly, the fluorescence signals of ICG and UCNPs exhibited similar distribution patterns (Figure S4a, Supporting Information), indicating a large overlap of membrane fluorescence signal from ICG and cargo fluorescence signal from UCNPs. The findings further implied satisfactory stability in vivo and efficient therapeutic agent delivery of PM‐PAAO‐UCNPs. In contrast, no selective accumulation was observed for the nude PAAO‐UCNPs or free photosensitizer Ce6 in the LCA or right carotid artery (RCA) (Figure S4b,c, Supporting Information), which might explain the low therapeutic efficacy of traditional photodynamic therapy.

**Figure 3:**
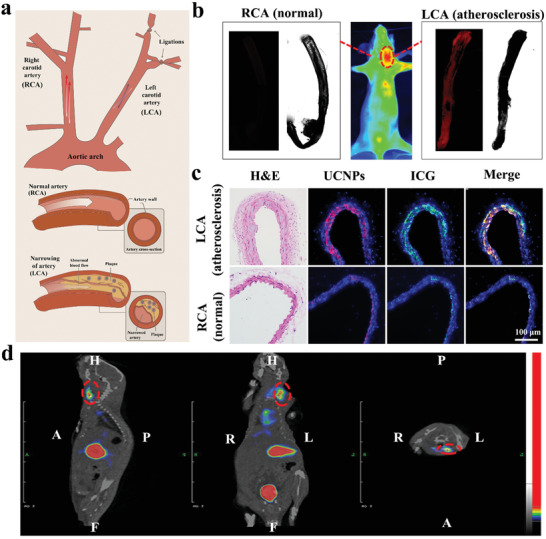
Targeting ability of PM‐PAAO‐UCNPs to atherosclerotic plaque in vivo. a) Schematic illustration of partial carotid ligation surgery model. The left carotid artery (LCA) was ligated and the right carotid artery (RCA) was used as control. b) Fluorescence images of mice injected with PM‐PAAO‐UCNPs (middle) and isolated arteries (left and right). c) H&E staining and fluorescent imaging of artery sections. Nuclei: blue; UCNPs: red; ICG: green; merge of ICG and UCNPs: yellow. d) SPECT/CT images of PM‐PAAO‐UCNPs (labeled with ^125^I) including transverse, coronal, and sagittal sections in model mouse 1 h following injection. Plaque region is denoted by the red circle.

Single‐photon emission computed tomography/computed tomography (SPECT/CT) was performed to further investigate the distribution and metabolism of PM‐PAAO‐UCNPs in vivo. As shown in Figure 3d and Figure S5 (Supporting Information), intravenous injection of PM‐PAAO‐UCNPs led to a prominent signal in atherosclerotic plaques in the LCA 30 min postinjection. The signal peaked at 1 h and was undetectable at 2 h postinjection. Consistent with the fluorescent imaging results, the radionuclide results also revealed accumulation of the radioactive signal of PM‐PAAO‐UCNPs in the LCA, but not in the RCA. In contrast, the nude PAAO micelle group exhibited only a marginal signal. These results suggested that this platelet‐mimic drug delivery system could accurately target atherosclerotic plaques in vivo, and therefore offer precise imaging of plaques and selective delivery of therapeutic payloads to plaque areas. In addition, precise imaging, especially SPECT/CT imaging, could be applied in the following guided phototherapy of plaque targets to improve the efficiency and quality of phototherapy and avoid unnecessary irradiation and side effects to normal tissues.

After confirming the delivery of targeted therapeutic agents, we sought to investigate the phototherapy efficacy of PM‐PAAO‐UCNPs in the intervention of atherosclerosis development. ROS production in macrophage‐derived foam cells was detected using dichlorfluorescein diacetate (DCFH‐DA) (Figure S6, Supporting Information). Upon 980 nm laser irradiation, a fluorescence signal of DCFH‐DA was detected, indicative of ROS production in foam cells. PM‐PAAO‐UCNPs generated ROS in concentration‐ and irradiation time‐dependent manners. To explore the penetration character of this UCNP‐based phototherapy system, a piece of thick muscle tissue was used to mimic living tissue for deep tissue PDT treatment. As shown in **Figure** **4**a, strong green fluorescence of DCFH‐DA was observed in foam cells under 660 or 980 nm irradiation. In contrast, control cells showed negligible ROS fluorescence signal. After blocking the excitation light with muscle tissue, the DCFH‐DA signal under 660 nm irradiation significantly decreased compared with that under 980 nm irradiation, suggesting that the 660 nm excitation wavelength of the photosensitizer Ce6 could hardly pass through the tissue to irradiate Ce6. Thus, ROS production was poor. In contrast, UCNP‐based phototherapy with 980 nm excitation could penetrate tissue and efficiently generate ROS in vivo.

**Figure 4:**
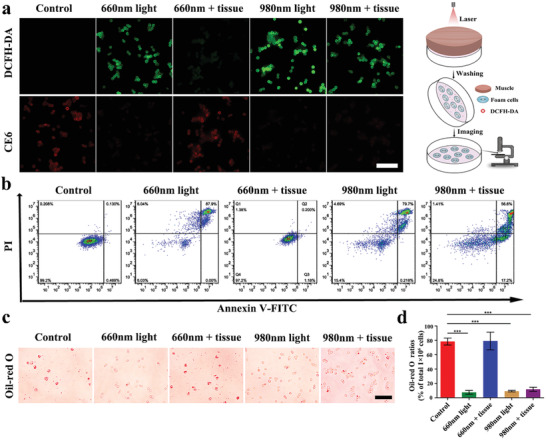
Deep tissue penetration of UCNPs‐based phototherapy in vitro. a) Detection of ROS in PM‐PAAO‐UCNP‐incubated foam cells using DCFH‐DA staining with 660 or 980 nm laser irradiation. One centimeter thick tissues were put on a cell‐culture dish to mimic in vivo condition (scale bar, 50 µm). Schematic illustration on the right shows the experimental procedure. b) Cell apoptosis detected using an Annexin V‐FITC/PI apoptosis detection kit after PDT treatment. c) The effect of PDT on lipid storage in foam cells measured with Oil Red O staining (scale bar, 50 µm). d) The ratio of Oil Red O stained cells in total 1 × 10^6^ cells. The results are presented as mean ± SD (*n* = 3). Statistical significance was evaluated using one‐way ANOVA. *p < 0.05, **p < 0.01, ***p < 0.001.

Moreover, PDT‐promoted ROS generation could induce the death of macrophage‐derived foam cells (Figure 4b; Figure S7a, Supporting Information). Consistent with increased ROS production, PDT using 980 nm wavelength irradiation significantly increased apoptosis of foam cells, even with thick muscle tissue coverage, owing to the deep tissue penetration of near‐infrared light and irreversible damage to foam cells by PDT. In addition, the reduction in cell viability after PDT was dependent on irradiation time and the photosensitizer dose. Notably, no obvious decrease in cell viability was observed with increasing concentrations of PM‐PAAO‐UCNPs, implying an acceptable biocompatibility of this platelet‐mimic system. Oil Red O staining (Figure 4c,d) revealed significantly decreased lipid storage in foam cells after 980 nm irradiation, indicating that PDT triggered lipid efflux from foam cells (Figure S7b, Supporting Information). These results suggested that PDT promotes cell apoptosis and lipid efflux of macrophage‐derived foam cells. The approach could be a promising therapeutic strategy to intervene in atherosclerosis by alleviating the lipid core composed of macrophage‐derived foam cells.

Based on the in vitro finding of the therapeutic efficacy of PDT, SPECT/CT imaging‐guided atherosclerosis phototherapy was conducted in a partial carotid ligation surgery mouse model. The model mice were intravenously injected with PM‐PAAO‐UCNPs and imaged with SPECT/CT to precisely identify the positions of atherosclerotic plaques at 30 min following injection. Then, laser irradiation at 980 nm was applied for the following photodynamic therapy in vivo. The results were compared with 660 nm laser irradiation. After two weeks of PDT therapy, LCAs were excised from the mice and sections were prepared for pathologic analysis. As shown in **Figure** **5**a, the nontreated group developed advanced atherosclerotic lesions in the LCA, as evidenced by an abundance of lipid cores and cholesterol clefts. In the 660 nm laser irradiation, PM‐PAAO‐UCNP‐treated group, the lipid content progressively increased and the plaque area gradually enlarged, reflecting unsatisfactory treatment efficacy caused by insufficient tissue penetration with the 660 nm laser and minimal ROS generation. In contrast, near‐infrared 980 nm laser irradiation achieved deep tissue penetration and sufficient ROS generation, which resulted in the significant reduction of LCA lipid deposition and mitigation of the further progression of atherosclerotic lesions. Serum cholesterol levels were tested at different time points after photodynamic therapy. The cholesterol concertation slightly increased at 7 and 14 d after 980 nm laser irradiation, and then restore to the original concentration before treatment (Figure S8, Supporting Information). The slightly increased concentrations were most likely induced by the efficient PDT‐promoted cell apoptosis and lipid efflux of foam cells after 980 nm laser irradiation. However, due to the high total serum cholesterol concentration in ApoE^−/‐^ mice model, this cholesterol concentration change was not obvious.

**Figure 5:**
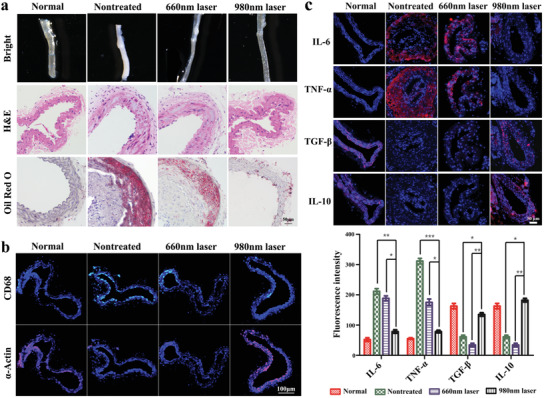
Radionuclide imaging‐guided atherosclerosis phototherapy in vivo. a) Bright field, H&E, and Oil Red O staining imaging of artery sections after radionuclide imaging‐guided PDT therapy (scale bar: 50 µm). b) Immunofluorescence imaging of CD68 and *α*‐Actin after PDT therapy (scale bar: 100 µm). c) Immunofluorescence imaging and fluorescence quantification of inflammatory cytokines (IL‐6, TNF‐*α*, TGF‐*β*, and IL‐10) in vascular slices (scale bar: 50 µm). Data are presented as mean ± SD (*n* = 6). Statistical analysis was performed via one‐way ANOVA. *p < 0.05, **p < 0.01, ***p < 0.001.

The observation that PDT induced foam cells to undergo apoptosis prompted the idea that the apoptosis of macrophage‐derived foam cells might be responsible for this lipid reduction in vivo. Immunofluorescence staining of macrophage marker CD68 confirmed that PDT induced macrophage‐derived foam cells to undergo apoptosis in vivo, and increased the expression of the vascular smooth muscle cell marker *α*‐actin. These findings indicated that the composition of atherosclerotic plaques changed after irradiation with 980 nm laser light (Figure 5b).^[^ ^19^ ^]^

Atherosclerosis is a chronic inflammatory disease and macrophages play a critical role in the inflammatory response. Hence, the elimination of macrophage‐derived foam cells by PDT might help reduce inflammation in atherosclerotic plaques. The expression levels of several inflammatory cytokines, including the proinflammatory cytokines interleukin‐6 (IL‐6) and tumor necrosis factor‐alpha (TNF‐*α*) and the anti‐inflammatory cytokines IL‐10 and transforming growth factor‐*β* (TGF‐*β*) were examined to evaluate the overall inflammatory status of the mice (Figure 5c).^[^ ^20^ ^]^ Compared with the untreated group and 660 nm treated group, IL‐6 and TNF‐*α* were significantly reduced in the 980 nm irradiation plaques, whereas IL‐10 and TGF‐*β* were substantially increased, indicating an amelioration of inflammation. Taken together, the efficient in vivo PDT with 980 nm laser irradiation could reduce lipid deposition in atherosclerotic lesions and change the atherosclerotic plaque environment from pro‐ to anti‐inflammatory, thus mitigating atherosclerotic plaque progression.

The lifetime of this platelet‐mimicking system was investigated. The elimination half‐life was calculated as 1.22 min for PAAO‐UCNPs and 7.45 h for PM‐PAAO‐UCNPs (**Figure** **6**a). As the PM coating can significantly prolong the plasma retention time of cargo, this strategy is particularly advantageous in treating blood vessel‐related disorders such as atherosclerotic plaques. The in vivo biodistribution of PM‐PAAO‐UCNPs was measured based on the quantification of Er^3+^, Y^3+^, and Yb^3+^ concentrations in various tissues using ICP‐MS (Figure 6b,c; Figure S9, Supporting Information). The two primary organs of the reticuloendothelial system (RES), the liver and spleen, contained the most nanoparticles. Furthermore, to evaluate the possible side effects of this platelet‐mimicking system, we performed tissue histology, complete blood count, and blood biochemistry analysis of mice treated with PM‐PAAO‐UCNPs. H&E staining and morphological imaging of major organs indicated no distinguishable injuries in all treated groups (Figure 6d,e), and no obvious body weight change was observed during the treatment period (Figure 6f). Complete blood count data indicated that the levels of red blood cells, white blood cells, platelets, and hemoglobin were in normal ranges in all treatment groups. In addition, blood biochemistry analysis showed that treatment with PM‐PAAO‐UCNPs had little influence on liver and kidney functions (Figure 6g). These results indicated that the platelet‐mimicking nanoparticles had relatively good biocompatibility, even at higher doses and with continuous administration. The influence of laser irradiation on skin and normal vessels was also tested with high‐intensity and long‐duration irradiation. Skin damage was only observed when the laser intensity increased to 50 mW cm^−2^ with 60 min of irradiation and slight changes were observed in all treated vessels (Figure S10, Supporting Information). These results demonstrate the safety and application potential of this therapeutic strategy. In clinical settings, the amount of radionuclide should be considered and explored to fulfill both imaging effectiveness and safety. In addition, near infrared laser irradiation in our experiment (10 mW cm^−2^, 30 min) was proved to be safe for skin and normal vessels, but higher laser power intensity and prolonged irradiation could induce skin damage. Thus, it is essential to control laser intensity, exposure area, and irradiation time.

**Figure 6:**
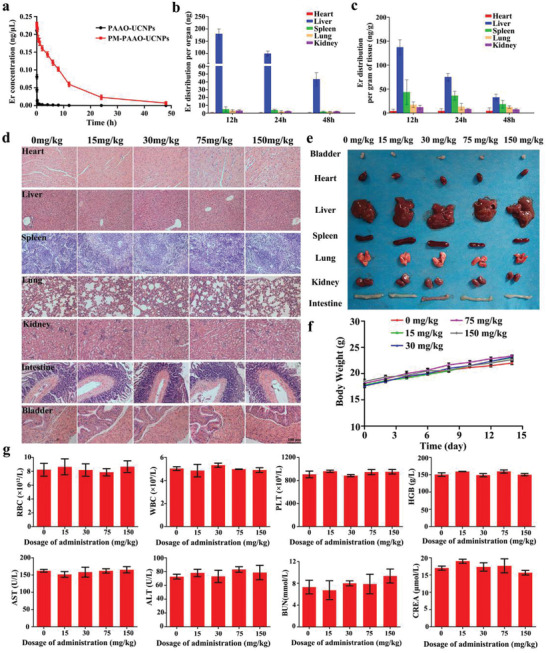
Systemic toxicity and circulation lifetime analysis of PM‐PAAO‐UCNPs. a) Systemic circulation lifetime analysis of PM‐PAAO‐UCNPs and PAAO‐UCNPs. Er^3+^ concentrations in the blood at 1, 2, 5, 10, 15, and 30 min and 1, 2, 4, 6, 8, 12, 24, and 48 h after administration were measured with ICP‐MS (mean ± SD, *n* = 6). b) Er^3+^ distribution per organ (mean ± SD, *n* = 6). c) Er^3+^ distribution per gram of tissue (mean ± SD, *n* = 6). d) Images of H&E staining sections and e) photographs of heart, liver, spleen, lung, kidney, intestine, and bladder. f) Mice body weights after administration of different doses of PM‐PAAO‐UCNPs during 14 d (mean ± SD, *n* = 6). g) Complete blood count and blood biochemistry analysis of mice 14 d after administration of different doses of PM‐PAAO‐UCNPs (mean ± SD, *n* = 6). Abbreviations are RBC: red blood cell; WBC: white blood cell; PLT: platelet; HGB: Hemoglobin; AST: aspartate transaminase; ALT: alanine transaminase; BUN: blood urea nitrogen; CREA: creatinine.

In this study, we successfully prepared a platelet‐mimicking delivery system for accurate localization and noninvasive PDT of atherosclerotic plaques. This strategy is based on the natural targeting ability of platelet to early‐stage plaques. The experiments have confirmed that the platelet‐mimicking nanoparticles could specifically accumulate in plaque sites rather than normal vessels. Radionuclide imaging with this system successfully distinguished plaques from normal vessels. More importantly, radionuclide imaging can be used to guide and control the irradiation spots during photodynamic therapy, thus realizing specific laser irradiation on plaque sites and avoiding possible damages to healthy surrounding tissues. Additionally, SPECT/CT‐guided PDT efficiently alleviates atherosclerosis progression by regulating lipid metabolism and reducing inflammation. This platelet‐mimicking delivery system may represent a potential noninvasive strategy to mitigate atherosclerotic plaque progression.

## Conflict of Interest

The authors declare no conflict of interest.

## Supporting information

Supporting Information
