# Supplementary material for: Platelet‐Mimicking Therapeutic System for Noninvasive Mitigation of the Progression of Atherosclerotic Plaques
Source: Adv Sci (Weinh). 2021 Feb 18;8(8):2004128. doi: 10.1002/advs.202004128 (PMC8061396; doi:10.1002/advs.202004128)
Supplement: Supplementary file 1 — Supporting Information [file ADVS-8-2004128-s001.pdf]

WILEY-VCH

© 2020 Wiley-VCH GmbH

ADVANCED SCIENCE
Open Access

Supporting Information

for *Adv. Sci.*, DOI: 10.1002/advs.202004128

Platelet-mimicking therapeutic system for noninvasive mitigating the progression of atherosclerotic plaques

Yi Ma, Yuxuan Ma, Mengqiu Gao, Zhihao Han, Wen Jiang, Yueqing Gu and Yi Liu**

Supporting Information

Platelet-mimicking therapeutic system for noninvasive mitigating the progression of atherosclerotic plaques

Yi Ma, Yuxuan Ma, Mengqiu Gao, Zhihao Han, Wen Jiang, Yueqing Gu, and Yi Liu**

Experimental Section

Preparation of PAAO-UCNPs. UCNPs were prepared by a modified solvothermal process according to the reported method. [1] YCl_3 (0.78 mmol), $YbCl_3$ (0.20 mmol) and $ErCl_3$ (0.02 mmol) and were mixed with 8 mL oleic acid (OA) and 15 mL octadecene (ODE) in a 50 mL flask. The solution was heated to 160 °C to form a homogeneous solution, and then cooled down to room temperature. 8 mL methanol solution containing NaOH (2.5 mmol) and NH_4F (4 mmol) was slowly added into the flask and stirred for 45 minutes. Subsequently, the solution was slowly heated and degassed at 120 °C for 30 minutes to remove methanol, and then heated to 300 °C and maintained for 1h under N_2 protection. After the solution was cooled naturally, nanoparticles were precipitated from the solution with ethanol, washed with ethanol/cyclohexane (9:1, v/v), and was separated by centrifugation (12000 rpm, 10 min) for three times to obtain white UCNPs. Then the UCNPs (20 mg) were dispersed in 10 mL chloroform, and mixed with amphiphilic micelle octylamine polyacrylic acid (PAAO, 40 mg). [2] After the mixture was sonicated for 5 min with a cell disrupter, 60 μL of Ce6 (10 mM) were added, stirred at room temperature overnight to remove chloroform. The residue was dispersed in deionized water to obtain the solution of PAAO-UCNPs. ”

Preparation of PAAO. The PPAO was prepared with poly(acrylic acid) (PAA, MW 3000) and n-octylamine.[2] NHS (0.48 g) and EDCI (0.8 g) were added to a solution of 0.6 g PAA in DMF (3 mL). After stirring for 6 h, DMF (1 mL) containing n-octylamine (233 μL) was

added to the above-mixed solution. After stirring for 12 h, the solution was purified by dialysis for 3 days (during which distilled water was changed every day).

Preparation of PM-PAAO-UCNPs Apolipoprotein E-knockout homozygous (ApoE$^{-/-}$) mice (18-20 g) were purchased from Laboratory Animal Resources of China Pharmaceutical University (Nanjing, China). All animal operation procedures were approved according to the Animal Care Guidelines of China Pharmaceutical University. Whole blood was collected from healthy ApoE$^{-/-}$ mice via the venous sinus. Platelets were isolated by differential centrifugation and the PM was derived using a repeated freeze-thaw process. The final PM-PAAO-UCNPs were fabricated by coating PM onto PAAO-UCNPs cores at a membrane protein to PAAO-UCNP weight ratio of 1:1 by sonicating in a bath sonicator.

The fluorescence signal quantification. The fluorescence imaging was performed by Leica DMi8 fluorescent microscope, and the average fluorescence intensity of specific areas could be directly quantified by Leica application suite X software. Areas in each cross section of atherosclerotic arteries were randomly selected.

***In vivo* adherence to atherosclerotic plaque.** ApoE$^{-/-}$ mice were used to perform partial carotid ligation surgery to model atherosclerosis. The mice were fed the high-fat (15%) Paigen diet (TP28900) for 2 weeks. Then, the atherosclerosis model mice received an intravenous injection of PM-PAAO-UCNPs labeled with ICG and ^{125}I. *In vivo* fluorescence imaging was performed by a modified CCD imaging system equipped with fluorescent filter sets and SPECT/CT.

***In vivo* PDT treatment.** PM-PAAO-UCNPs (15 mg/kg) were administered to ApoE$^{-/-}$ mice via tail vein injection 30 min before light administration. Under anesthesia, the LCAs of mice were irradiated with a 980 nm laser (10 mW cm^{-2}) for 30 min with a 660 nm laser (10 mW cm^{-2}) treatment as a control (n=6). The PDT treatment was performed every 2 days. Fourteen days after the PDT treatment, the mice were sacrificed and the LCA and RCA were harvested from each mouse. Transverse carotid sections (10 μm thick) were collected and stained with

Oil Red O, H&E, and 4',6-diamidino-2-phenylindole (DAPI), or were exposed to antibody to CD68, IL-6, IL-10, TNF-α, TGF-β, and α-Actin followed by secondary antibody coupled to a fluorescent probe. Fluorescence imaging was performed using a model DMi8 fluorescent microscope (Leica, Wetzlar, Germany).

Half-life and biodistribution of PM-PAAO-UCNPs in blood PM-PAAO-UCNPs were injected into $ApoE^{-/-}$ mice via the tail vein. Blood and major organs were collected and immersed in 3 mL of 60% ultrapure nitric acid and incubated at 110°C on an oil bath pan until the blood was completely digested. The Er concentration was measured using ICP-MS. The elimination half-life was determined by fitting the experimental data using the Phoenix WinNonlin version 8.1 software (Certara, Princeton, NJ) and a two-compartment model.

Statistical analysis. All experiments were independently replicated at least three times. Analysis of variance (ANOVA) was used to analyze differences between groups (GraphPad Prism). The results are presented as mean ± standard deviation (SD). Asterisk (*) denotes statistically significant differences (*$p < 0.05$, **$p < 0.01$, ***$p < 0.001$).

Reference:

[1] Y. Liu, M. Chen, T. Cao, Y. Sun, C. Li, Q. Liu, T. Yang, L. M. Yao, W. Feng, F. Li, *J. Am. Chem. Soc.* **2013**, 135, 26, 9869–9876.

[2] M. Cui, H. Li, X. Ren, L. Xia, D. Deng, Y. Gu, D. Li, P. Wang, *Nanoscale* **2020**,12, 17517-17529.

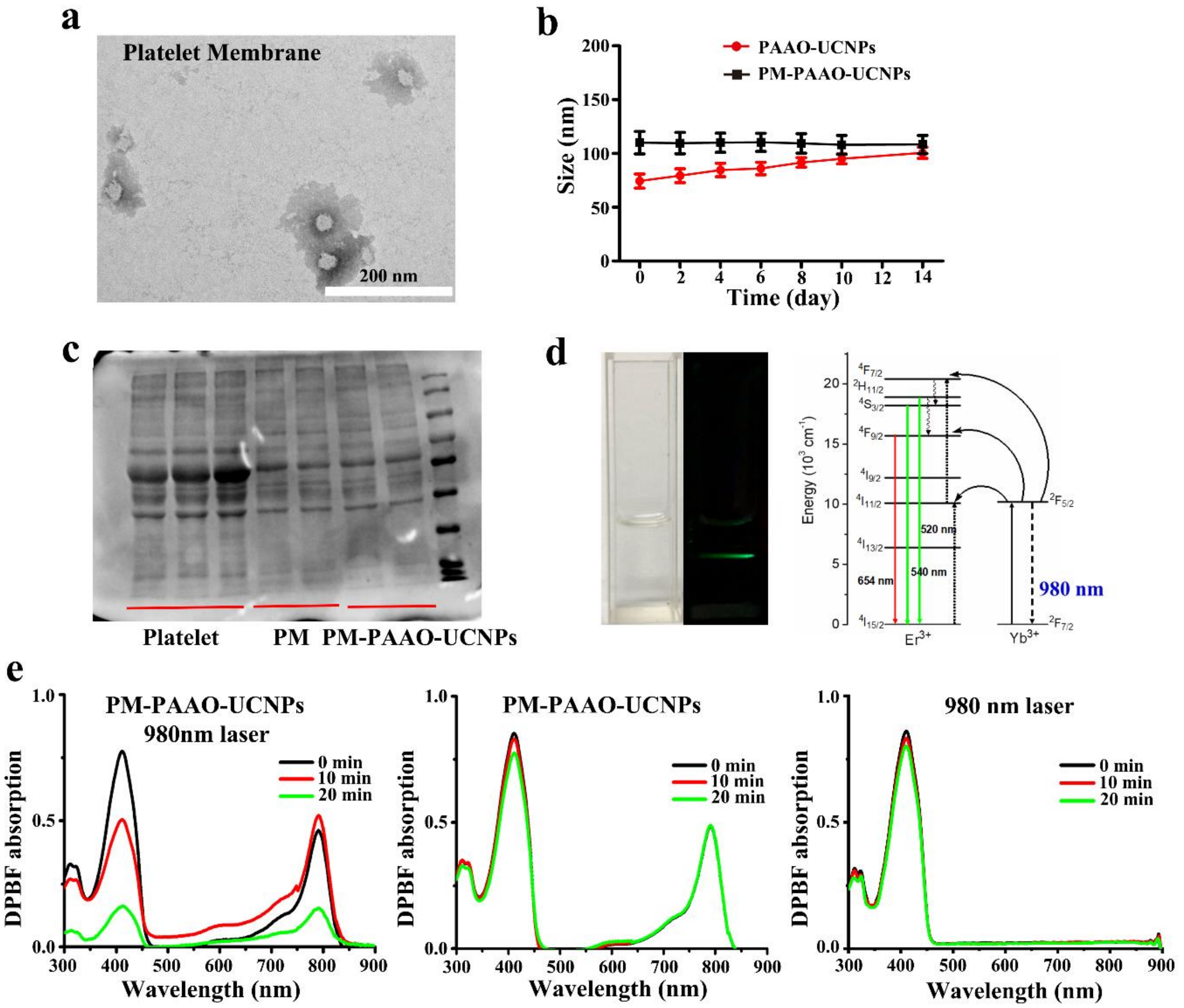

Figure S1. Characterization of platelet membrane (PM), PAAO-UCNPs, and PM-PAAO-UCNPs. a) Transmission electron microscopy image of PM. b) Size change of PAAO-UCNPs and PM-PAAO-UCNPs in PBS for 14 days. Data are presented as mean ± SD (n = 3). c) Protein content analysis of platelet, PM, and PM-PAAO-UCNPs using Coomassie blue staining. d) The photograph and mechanism of fluorescence emission in UCNPs under 980 nm laser excitation. e) Absorption spectra of the 1,3-diphenylisobenzofuran (DPBF) probe in different conditions at 0, 10, and 20 min.

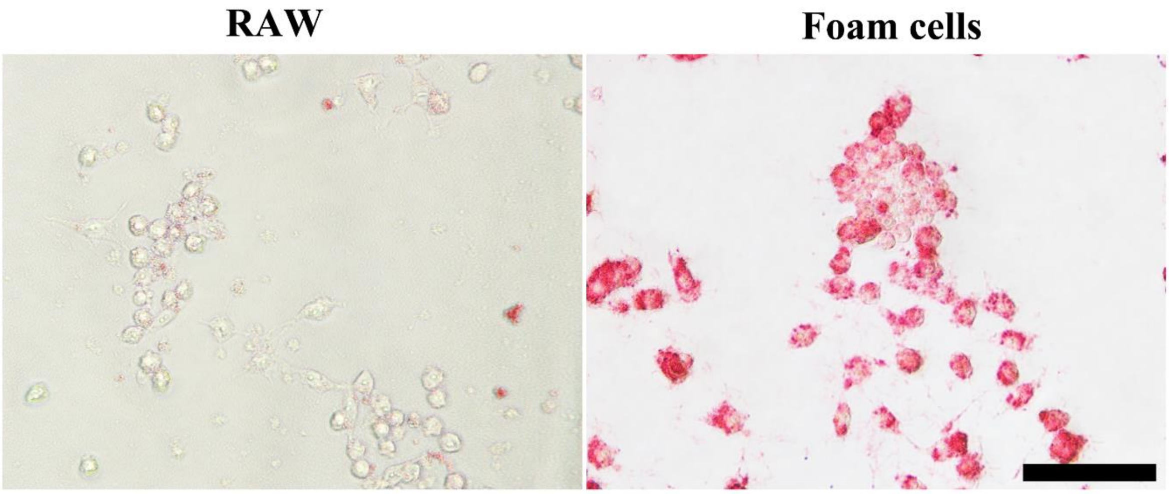

Figure S2. Oil Red O staining images of RAW and foam cells (scale bar, 50 μm).

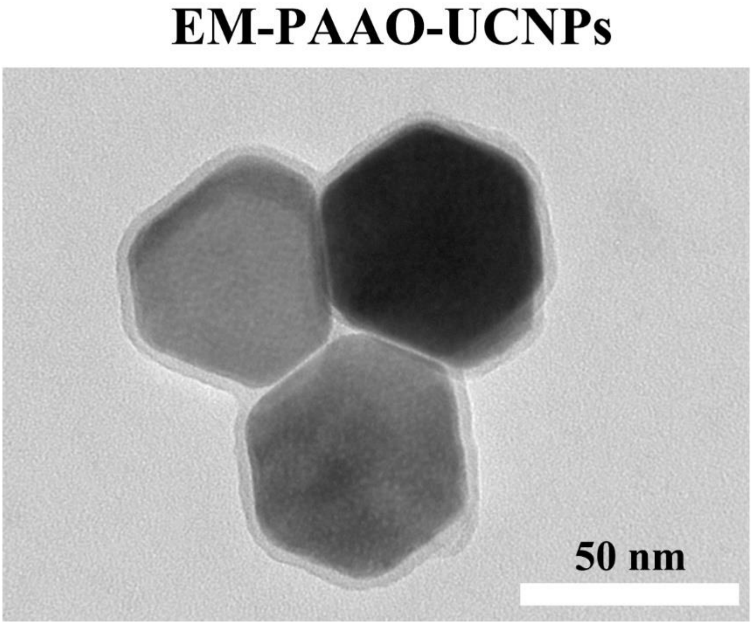

Figure S3. TEM image of erythrocyte membrane-coated UCNPs (EM-PAAO-UCNPs).

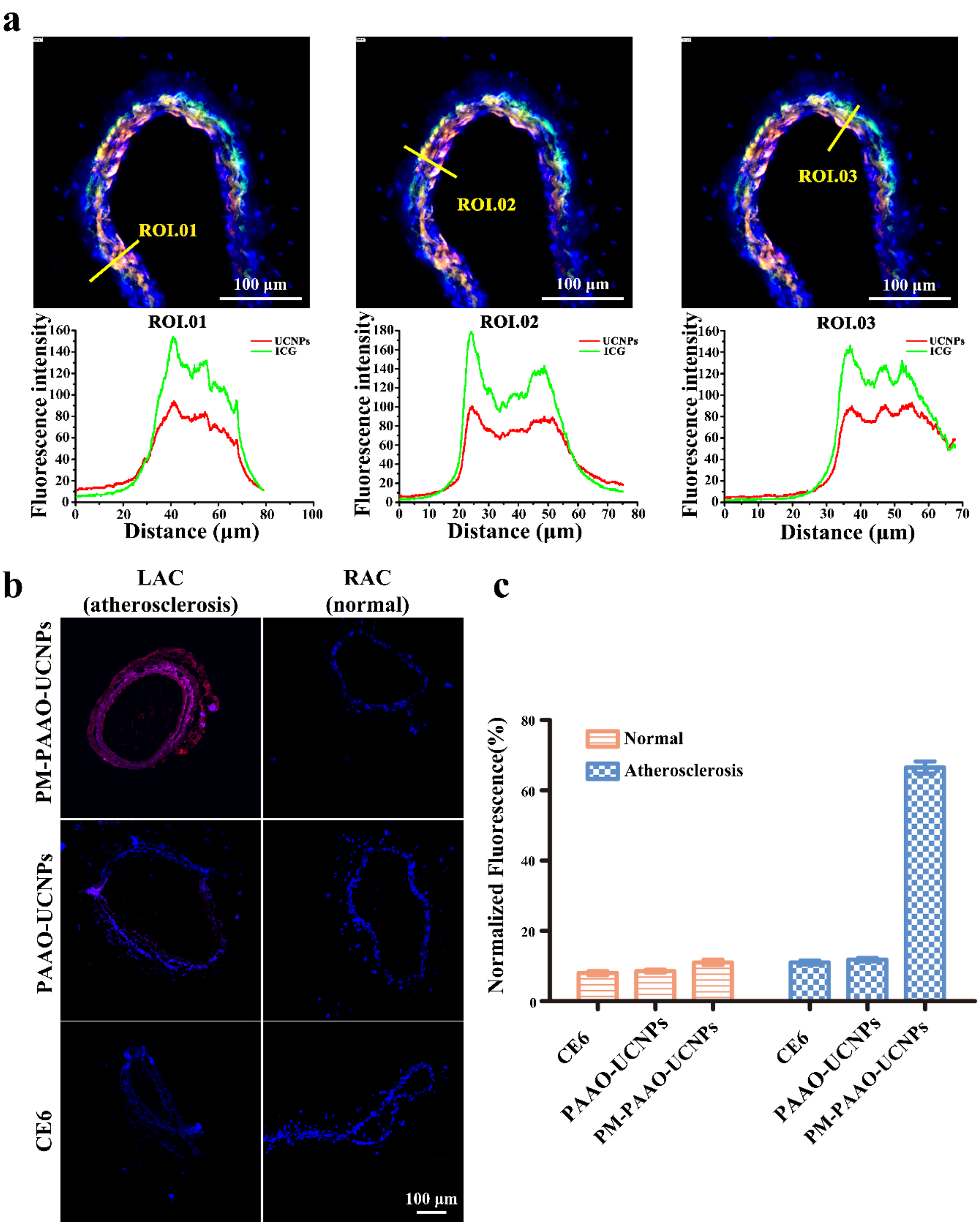

Figure S4. Fluorescent images of histological cross section of atherosclerotic arteries. a) Two-channel overlap of UCNPs and ICG in artery sections. A linear profile was used to characterize the overlap degree of red fluorescence from UCNPs and green fluorescence from ICG. b) Fluorescent imaging of artery sections after administration of PM-PAAO-UCNPs, PAAO-UCNPs and CE6 (nucleus: blue; CE6: red). c) Normalized fluorescence of artery sections (mean ± SD, n = 6).

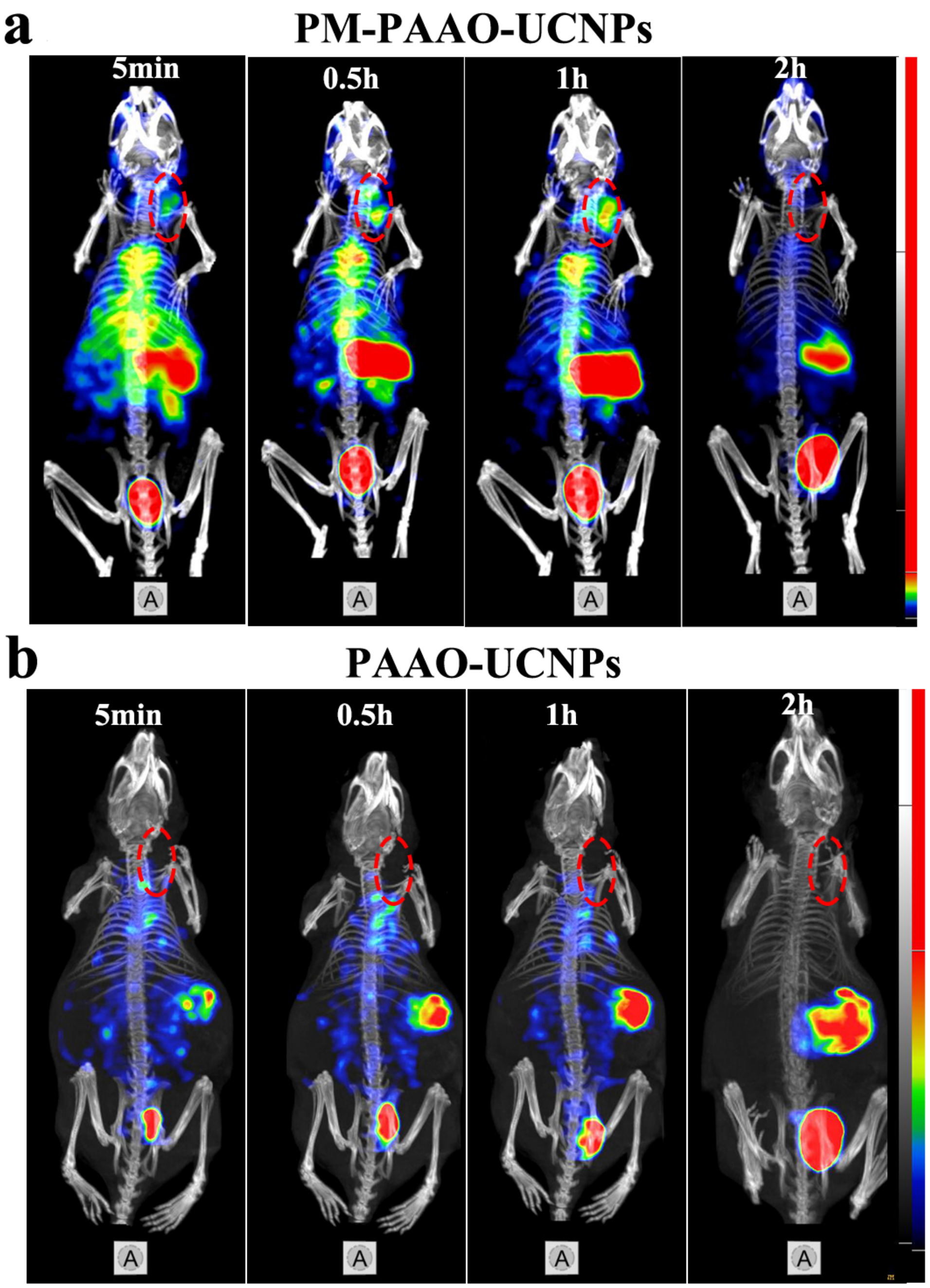

Figure S5. SPECT/CT images of partial carotid ligation surgery mouse model after administration of I125 labeled PM-PAAO-UCNPs (a) and PAAO-UCNPs (b) at different times (5 min, 0.5 h, 1 h, and 2 h). Plaques are denoted by the red circle.

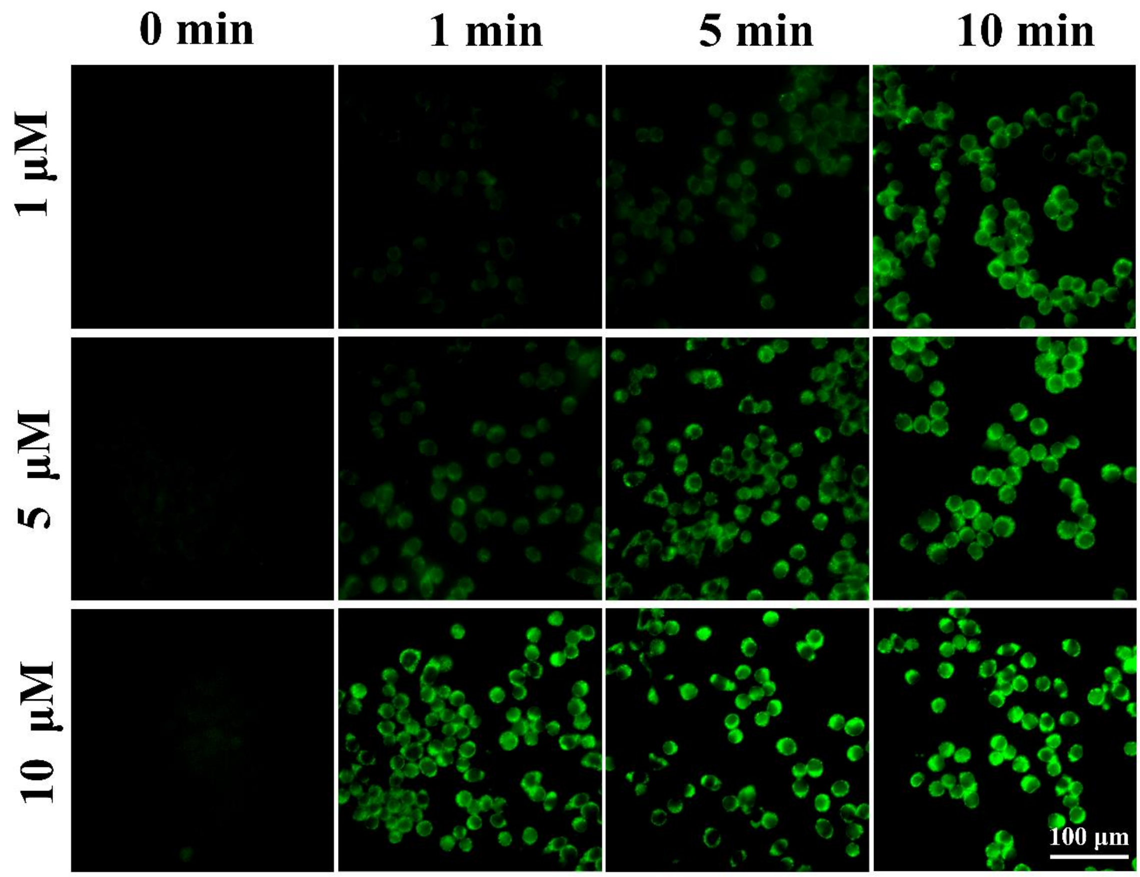

Figure S6. Detection of intracellular ROS in foam cells incubated with PM-PAAO-UCNPs at different concentrations (1, 5, and 10 μM) for different irradiation times (0, 1, 5, and 10 min) by DCFH-DA staining.

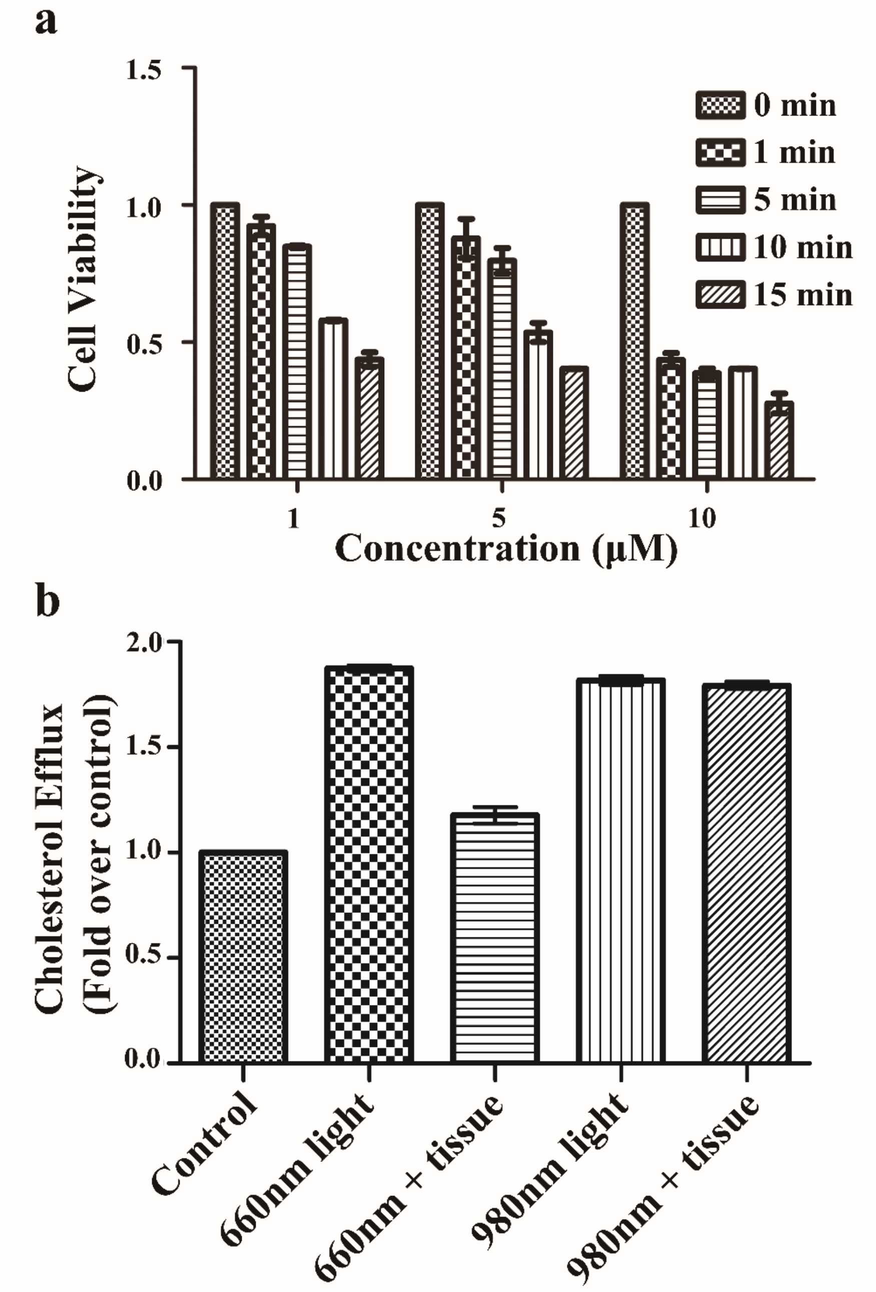

Figure S7. Cell viability and cholesterol efflux of foam cells after PDT treatment. a) Cell viability of foam cells incubated with different concentrations (1, 5, and 10 µM) of PM-PAAO-UCNPs for different irradiation times (0, 1, 5, 10, and 15 min). Results are expressed as mean ± SD (n=3). b) Fluorometric assay test of cholesterol efflux from foam cells after PDT treatment. Results are expressed as mean ± SD (n=3).

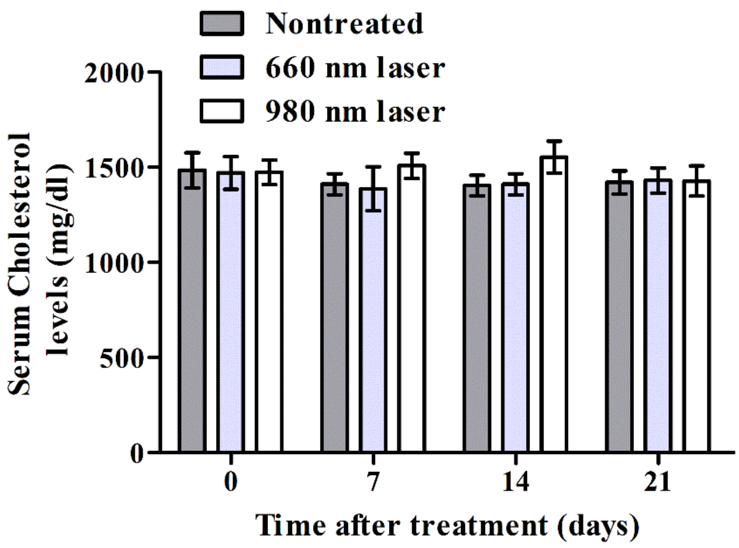

Figure S8. Serum cholesterol levels at different time points after photodynamic therapy.

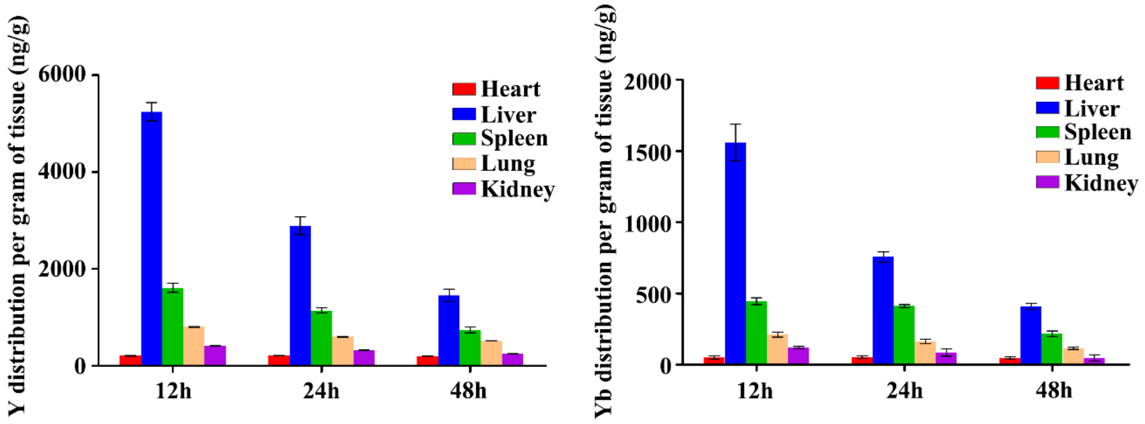

Figure S9. Y and Yb distribution data per gram of tissue.

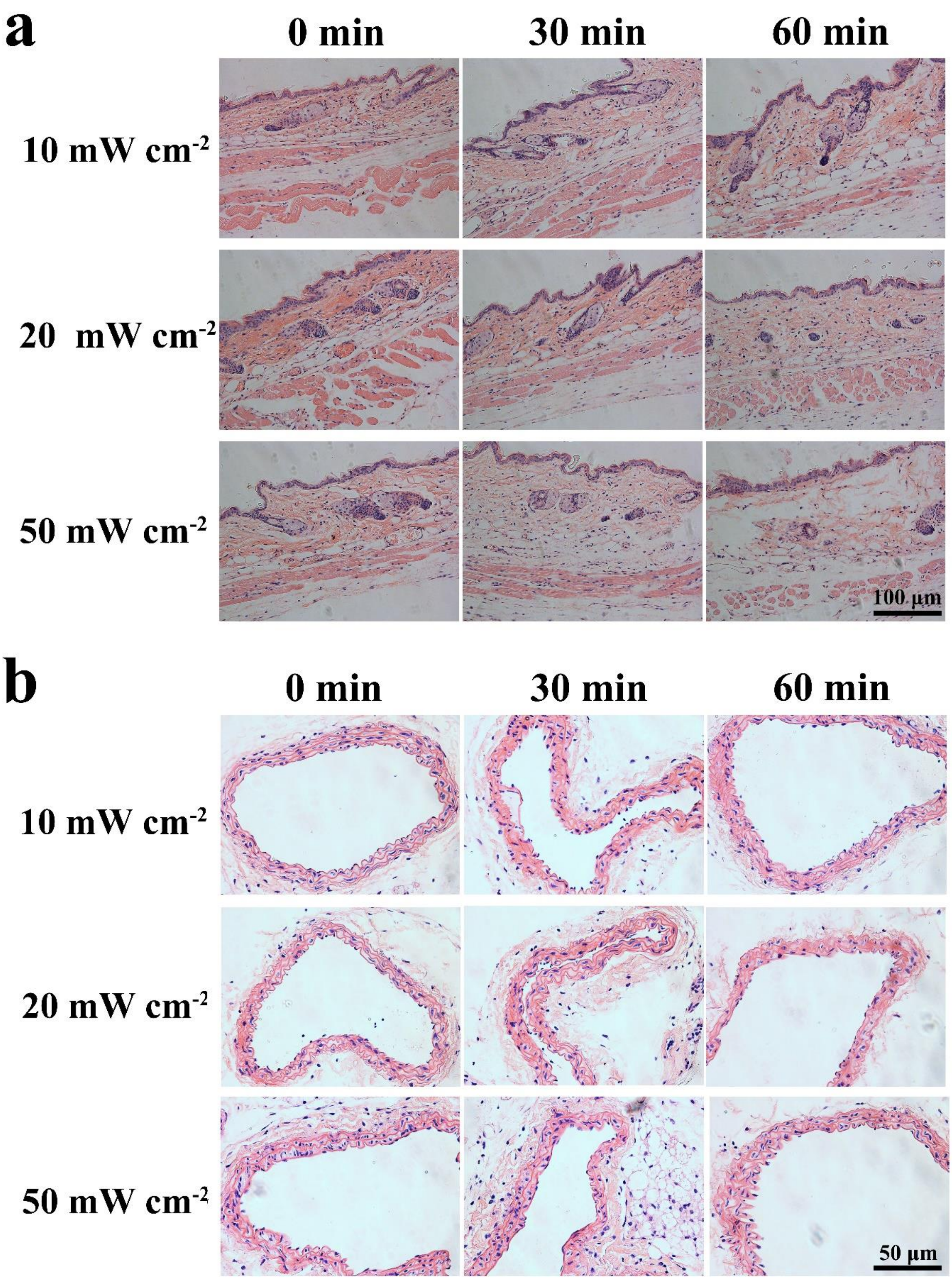

Figure S10. H&E stain images of skin sections (a) and blood vessels (b) using different laser strengths (10, 20 and 50 mW cm^{-2}) for different irradiation times (0, 30, and 60 min).
